# Supplementary material for: Dose response comparison of Nipah virus strains Malaysia and Bangladesh in hamsters exposed by the intranasal or intraperitoneal route
Source: PLoS One. 2025 May 12;20(5):e0318912. doi: 10.1371/journal.pone.0318912 (PMC12068590; doi:10.1371/journal.pone.0318912)
Supplement: S1 Data — (PDF) [file pone.0318912.s001.pdf]

<u>Supporting Information, Figures 1-2</u>

Survival - NiV-B

Study Day	10^2 pfu IN	10^3 pfu IN	10^4 pfu IN	10^5 pfu IN	10^6 pfu IN	10^4pfu IP	10^5pfu IP	10^6pfu IP	10^7pfu IP
1	0	0	0	0	0	0	0	0	0
1	0	0	0	0	0	0	0	0	0
1	0	0	0	0	0	0	0	0	0
1	0	0	0	0	0	0	0	0	0
1	0	0	0	0	0	0	0	0	0
1	0	0	0	0		0	0	0	0
1	0	0	0	0					
1	0	0	0	0					
1				0					
1				0					
1				0					
1				0					
1				0					
2									
3									1
4									1
5								1	1
5								1	1
6	1					1		1	
7						1	1		
7						1	1		
8									1
9							1	1	
10					1	1			
11					1		1	1	
11									
11									
12					1				
13	1						1		

13							1		
14									
15									1
15									
15									
15									
15									
15									
15									
15									
16									
16									
16									
16									
16									
16									
16									
16									
17	0	0	0	0	0	0		0	
17	0	0	0	0	0	0			
17	0	0	0	0					
17	0	0	0	0					
17	0	0	0	0					
17	0	0	0	0					
17		0	0	0					
17		0	0	0					
17				0					
17				0					
17				0					
17				0					
17				0					

Survival - NiV-M

Study Day	10^6 pfu IN	10^5 pfu IN	10^4 pfu IN	10^3 pfu IN	10^7 pfu IP	10^6 pfu IP	10^5 pfu IP	10^4 pfu IP
1	0	0	0	0	0	0	0	0
1	0	0	0	0	0	0	0	0
1	0	0	0	0	0	0	0	0
1	0	0	0	0	0	0	0	0
1	0	0	0	0	0	0	0	0
1	0	0	0	0	0	0	0	0
2								
3								
4					1			
4					1			
5					1	1		
5					1	1		
6						1	1	
6							1	
6	1						1	
7					1	1	1	1
7					1			
7								
8						1		1
8							1	1
9		1					1	
9								
10	1							
10	1							
10	1							
10	1							
10	1							
11		1						
11								

11					
11					
11					
12					
13			1		
14					
15					
16					
17	0	0	0	0	0
17	0	0	0		0
17	0	0	0		0
17	0	0	0		
17		0	0		
17		0			

Supporting Information, Figure 3

Survival - NiV-B

Study Day	NiV_B Controls	NiV_B 600/300 mg/kg	NiV_B 300/150 mg/kg
0	0	0	0
0	0	0	0
0	0	0	0
0	0	0	0
0	0	0	0
0	0	0	0
0	0	0	0
0	0	0	0
1			
2			
3			
4			
5	1		
5	1		
5			
6	1		1
6	1		
6	1		
6	1		
7			1
7			1
7			
8		1	
8		1	
9	1		
10			
11			
12		1	

12		1	
13			
14			
15			
16			
17			
18			
19			
20			1
21	0	0	0
21		0	0
21		0	0
21		0	0

Survival - NiV-M

Study Day	NiV_M Controls	NiV_M 600/300 mg/kg	NiV_M 300/150 mg/kg
0	0	0	0
0	0	0	0
0	0	0	0
0	0	0	0
0	0	0	0
0	0	0	0
0	0	0	0
0	0	0	0
1			
2			
3			
4	1		
5	1		1
5	1		
5	1		
6	1		1

6			1
6			
6			
7	1	1	1
7		1	
7		1	
8			
8			
9			
10			1
11		1	
12	1		
12			
13			
14			
15			1
16			
17			
18			
19			
20			
21	0	0	0
21		0	0
21		0	
21		0	

Supporting Information, Figure 4

			Controls					
NiV-B	1	12	12	13	13	12	12	12
NiV-M	12	15	1	9	15	12	12	13
			600/300 mg/kg					
NiV-B	12	12	0	2	15	2	1	8
NiV-M	8	1	1	15	1	13	12	1
			300/150 mg/kg					
NiV-B	12	9	3	12	1	11	0	2
NiV-M	12	15	0	15	8	15	15	0

Supporting Information, Figure 5

Group Data - NiV-B

Controls

0	0	0	0	0	0	0
6	0		2.95	4.69	5.27	
21	0					

600/300 mg/kg

0	0	0	0	0	0	0
6	0	0	0	0	0	1.62
21			2.54	1.8		2.54

300/150 mg/kg

0	0	0	0	0	0	0
6	0	0	1.93	0	0	0
21			0		0	

Group Data - NiV-M

Controls

0	0	0	0	0	0	0
6		1.39	2.14		5.93	4.15
21			2.74			

600/300 mg/kg

0	0	0	0	0	0	0
6	1.79	1.71	0	0	2.02	0
21		2.11	1.72		2.56	

300/150 mg/kg

0	0	0	0	0	0	0
6	1.83	4.21	0	0	2.13	2.19
21			2.14			

Peak Data Analysis

			Controls			
NiV-B	0	2.95	4.69	5.27		
NiV-M	1.39	2.74	5.93	4.15		
			600/300 mg/kg			
NiV-B	0	0	2.54	1.8	0	2.54
NiV-M	1.79	2.11	1.72	0	2.56	0
			300/150 mg/kg			
NiV-B	0	0	1.93	0	0	0
NiV-M	1.83	4.21	2.14	0	2.13	2.19
